# Bioinformatics analysis combined with experimental validation reveals the novel mechanisms of multi-targets of dapagliflozin attenuating diabetic liver injury

**DOI:** 10.3389/fendo.2025.1519153

**Published:** 2025-05-12

**Authors:** Pengyu Wang, Zhen Sun, Qing Lan, Shuo Zhang, Yan Song, Leiming Yang, Mi Chen, Jianfen Shen, Qi Huang, Youzhi Zhang

**Affiliations:** ^1^ Hubei Key Laboratory of Diabetes and Angiopathy, School of Pharmacy, Hubei University of Science and Technology, Xianning, China; ^2^ Hubei Engineering Research Center of Traditional Chinese Medicine of South Hubei Province, Hubei University of Science and Technology, Xianning, China; ^3^ School of Medicine, Shanghai Jiao Tong University, Shanghai, China; ^4^ Experimental Animal Center, Guangzhou Women and Children’s Medical Center, Guangzhou Medical University, Guangzhou, Guangdong, China; ^5^ Department of Central Laboratory, The Affiliated Hospital of Jiaxing University, Jiaxing, Zhejiang, China

**Keywords:** dapagliflozin, diabetic liver injury, ERK/IKKβ/NF-κB pathways, bioinformatics, data mining, metabolic disease

## Abstract

**Objective:**

Diabetic liver injury, a chronic complication of diabetes mellitus (DM), has been extensively documented. Dapagliflozin, a sodium-glucose co-transporter 2 (SGLT2) inhibitor, has shown significant therapeutic benefits in clinical trials for the management of diabetes However, the specific mechanism on the treatment of diabetic liver injury with dapagliflozin is not fully understood. Therefore, this study aims to further explore the potential mechanism of dapagliflozin on diabetic liver injury based on bioinformatics analysis and experimental verification.

**Methods:**

Diabetic liver injury was induced by a high-fat diet combined with STZ in mice. Biochemical kit detection and H&E staining were used to observe lipid aggregation and oxidative stress in liver tissue. Moreover, the expression of inflammatory and apoptosis-related factors was detected using western blotting (WB) and quantitative polymerase chain reaction (qPCR). Subsequently, differential expressions genes analysis, weighted gene co-expression network analysis (WGCNA), molecular docking, as well as molecular dynamics was conducted based on the Gene Expression Omnibus (GEO) and pharmacology databases. Finally, WB and qPCR were performed to validate the mechanism of dapagliflozin on diabetic liver injury *in vivo* and *in vitro*.

**Results:**

Dapagliflozin alleviated diabetic liver injury by decreasing lipid deposition, oxidative stress levels, the inflammatary and apoptosis-related proteins and mRNA levels, while it also reducing blood glucose. Mechanically, 78 overlapping genes of dapagliflozin and diabetic liver injury were obtained. Notably, *Mapk3*, *Mapk1*, *Ikbkb*, and *Nfkb1* as the hub genes involved in dapagliflozin attenuating diabetic liver injury were identified, and dapagliflozin exhibited better affinity with these proteins. Moreover, dapagliflozin inhibited the elevated protein (genes) levels of ERK1/2 (*Mapk3, Mapk1*), IKKβ(*Ikbkb)*, and NF-κB (*Nfkb1*), which are induced by diabetic liver injury, as confirmed by both *in vivo* and *in vitro* experiments.

**Conclusion:**

Dapagliflozin ameliorated diabetic liver injury by inhibiting the ERK/IKKβ/NF-κB signalling pathway, as demonstrated by bioinformatics analysis combined with *in vivo* and *in vitro* experiments.

## 1 Introduction

Diabetes mellitus (DM) is a metabolic disorder that characterized by insulin resistance and increased blood glucose (1, 2). The increased incidence of chronic complications associated with diabetes has emerged as the primary contributor to mortality and disability resulting from this condition (3). Research has shown that liver damage, including non-alcoholic fatty liver disease, hepatitis, and cirrhosis, occurs in more than 50% of diabetic patients (4). When the long-term chronic elevation of blood glucose exceeds the clearance capacity of the liver, it causes oxidative stress and abnormal secretion of inflammatory cytokines, leading to impaired liver function in diabetes (5). However, despite extensive research efforts on diabetic liver injury, the underlying mechanisms responsible for its development remain incompletely understood, and there is currently a lack of identified therapeutic targets and pharmacological interventions.

Dapagliflozin is a novel antidiabetic medication that inhibits sodium-glucose cotransporter 2 (SGLT2). It has exhibited efficacy in the reduction of urine protein excretion, the promotion of weight loss, and decreased blood glucose levels (6). Consequently, dapagliflozin has gained significant traction in clinical practice and has been extensively employed (7). Meanwhile, according to reports, dapagliflozin has the potential to alleviate the advancement of non-alcoholic fatty liver disease and liver fibrosis by reducing the inflammation and oxidative stress resulting from hyperglycemia (8–10). However, it is not partially unknown how dapagliflozin treats diabetic liver damage caused by STZ. Therefore, the mechanism by which dapagliflozin reduces diabetic liver damage will be the focus of this study, which will provide a theoretical direction for future clinical applications.

The extracellular signal-regulated kinase (ERK) signalling pathway, a crucial component of the MAPK cascade, is acknowledged for its involvement in regulating oxidative stress and inflammation (11, 12). Within, it played a crucial role in various biological processes, including maintaining cell morphology, differentiation, and proliferation (13, 14). Modern pharmacological research indicated that the investigations of ERK1/2 focus on cancer-related studies, with a primary emphasis on the mechanism related to the activation of apoptosis in cancer cells (15–17). Moreover, Nuclear transcription factor kappa B (NF-κB) is widely recognized as a critical modulator of inflammation (18). It was demonstrated that liver damage was closely related to the activation of inflammatory factors (IL-1β, IL-6, and IL-18) (19). Ginsenoside Rg1 mitigates acute liver injury by down-regulating the protein levels of NF-κB and diminishing the production of IL-6 and IL-18 (20). At the same time, β-sitosterol can suppress the IKKβ/NF-κB signalling pathway and improve inflammation in adipose tissue, thereby inhibiting insulin resistance caused by obesity (21). Therefore, it would be worth exploring whether inflammation induced by the NF-κB signalling pathway is associated with diabetic liver injury. Recent studies have shown that gastrin/CCKBR alleviates type 2 diabetes by inhibiting SGLT2-mediated glucose reabsorption through the ERK/NF-κB signalling pathway (22). Notably, there are currently few reports elucidating the relationship between dapagliflozin and the ERK/IKKβ/NF-κB in diabetic liver injury, and it will be extremely valuable in the treatment of diabetic liver injury with dapagliflozin.

Collectively, this study was divided into three sections. First, the related indicators of the high-fat diet combined with STZ diabetic liver injury mice were detected, including lipid accumulation, oxidative stress, inflammatory factors, apoptosis-related protein and mRNA expression. Subsequently, the potential targets of dapagliflozin treating diabetic liver injury were identified based on analysis by the Gene Expression Omnibus (GEO) and pharmacology databases. Finally, the potential mechanism of how dapagliflozin improves diabetic liver injury was verified *in vivo* and *in vitro*. The findings of this study contribute to the advancement of dapagliflozin in clinical application by providing a solid theoretical foundation.

## 2 Materials and methods

### 2.1 Materials

Dapagliflozin (Dapa, Cat# D126800) and streptozotocin (STZ, Cat# S110910) were purchased from Shanghai Aladdin Biochemical Technology Co., Ltd. Roswell Park Memorial Institute 1640 (RPMI-1640, Gibco, 72400047) and Fetal Bovine Serum (FBS, Gibco, 10099-141) were purchased from Thermo Fisher Scientific (Shanghai) Co., Ltd. HL-7702 cells (Cat# CL-0190) were purchased from Wuhan Pricella Biotechnology Co., Ltd. The concentrations of GSH (Cat# A005-1-2), MDA (Cat#A003-1-2), CAT (Cat#A007-1-1),and SOD (Cat#A001-3-2), AST (C010-2-1), ALT (C009-2-1), TG (A110-1-1), T-CHO (A111-1-1) were purchased from Nanjing Jiancheng Bioengineering Institute, Nanjing, China. The primary antibodies including p-ERK1/2 (CST, Cat#4370S, 1:1000), ERK1/2 (CST, Cat#9120S, 1:1000), IKKβ (Wanleibio, Cat#WL04340, 1:1000), Cleaved PARP1 (Proteintech, Cat#60555-1-Ig, 1:1000), TNF-α (ABclonal, Cat#A11534, 1:1000), p-NF-κB p65 (Abcam, Cat#ab86299, 1:1000), NF-κB p65 (Bioworld, Cat#bs1253 1:1000), SGLT2 (Biodragon, Cat#BD-PT4274, 1:1000) BAX (ABclonal, Cat#A15646, 1:1000), BCL2 (Bioss, Cat#bs-0032R, 1:1000), Cleaved-caspase3 (CST, Cat#9661S, 1:1000), Caspase3 (CST, Cat#9662, 1:1000), IL-6 (ABclonal, Cat#A0286, 1:1000), IL-18 (ABclonal, Cat#A20473, 1:1000), and IL-1β (SAB, Cat#41059-2, 1:1000), GAPDH (Cat#LF205; Epizyme, shanghai, 1:5000), β-actin (Cat#LF201; Epizyme, shanghai, 1:10000), α-tubulin (Cat#LF210; Epizyme, shanghai, 1:10000) were used for western blotting.

### 2.2 Animal and experimental procedure

**Figure 1:**
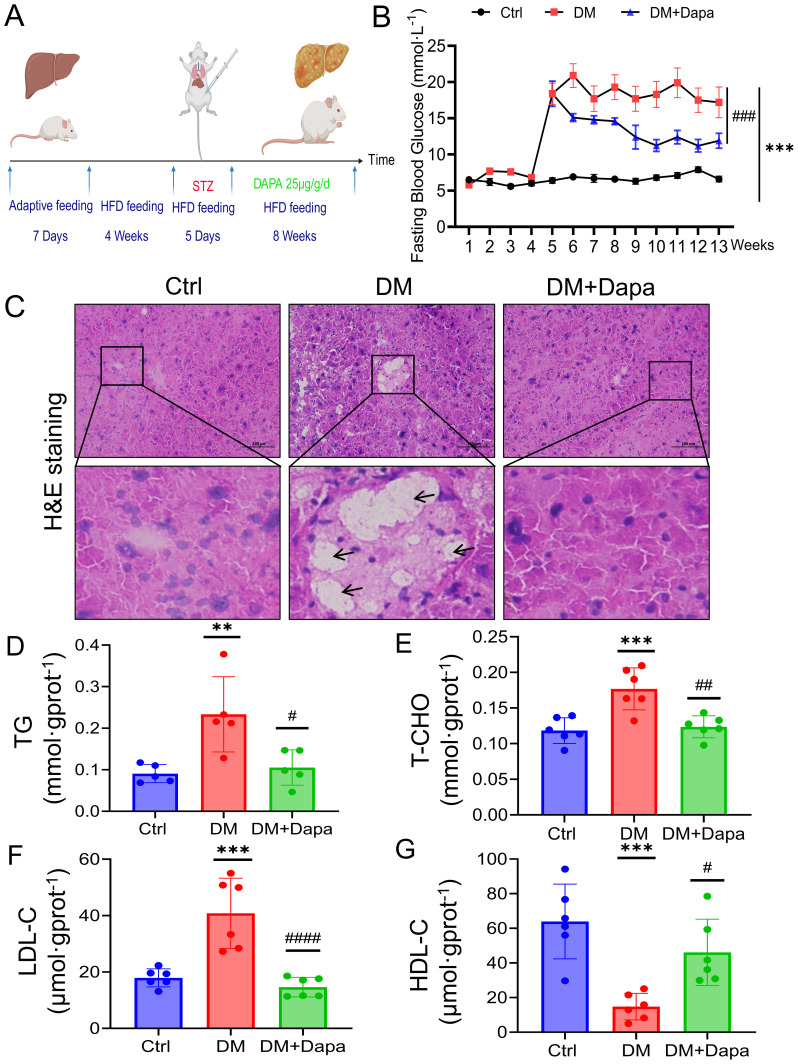
Dapagliflozin protects against diabetic liver injury by attenuating lipid accumulation. **(A)** Flowchart of the feeding and harvesting of mice. All the mice were allowed to acclimate for 7 days, and the diabetes mellitus (DM) and DM+Dapa groups were fed a high-fat diet for 4 weeks before STZ injection (5 days). The mice were treated with dapagliflozin for 8 weeks. **(B)** Dapagliflozin reduced fasting blood glucose levels, which had significantly increased to approximately 20 mmol/L in the DM group of mice following 5 days of STZ injections and dapagliflozin could remarkly reduce the glycemia of the mice with diabetes to approximately 10 mmol·L^-1^. n = 10–20. **(C)** H&E staining was performed on mouse livers. The black arrows and red arrows represent lipid vacuolization and lipid accumulation, respectively. **(D–G)** The effects of dapagliflozin on TG, T-CHO, HDL-C, and LDL-C in the mouse liver (n≥5). ^**^P < 0.01, ^***^ *P < 0.001* vs. the Ctr group; ^#^ *P < 0.05*, ^##^ *P < 0.01*, ^###^ *P < 0.001* vs the DM group.

50 male C57BL/6 mice, aged 6–8 weeks and weighing 25 ± 2 g were raised in the SPF animal room. All animal experimental techniques have been supported by The Animal Care and Use Committee of Hubei University of Science and Technology (IACUC Number: 2021-05-120). The mice were kept in a room with consistent humidity of 50 ± 5% and an ambient temperature of 22 ± 2°C (three to five per cage). The mice were exposed to a 12-hour light-dark cycle. After an adaptation period of one week, the mice were randomly assigned at random to two groups: the Ctr, and DM groups. The control group was provided with standard feed, while the DM group was fed a high-fat diet consisting of 66.5% standard feed, 13.5% lard, and 20% sucrose. Following a 4-week period, DM group were randomly divided into the DM group and the DM+Dapa groups, all animals had an overnight fasting. The DM and DM+Dapa group were then injected intraperitoneally with 40 mg/kg STZ (5 days), which was dissolved in a 0.1M citrate buffer with a pH of 4.5. The Ctr group received an injection of the same quantity of citric acid salt buffer. The DM and DM+Dapa groups mice were fed a high-fat diet for another 8 weeks. After the modelling, the DM+Dapa group was given drinking water containing dapagliflozin 25μg/g/d, and the rest drank normal water for a total of 8 weeks (**Figure 1A**), and fasting blood glucose was assessed once a week (**Figure 1B**).

### 2.3 Cell culture and cell viability assay

HL-7702 cells were cultured in RPMI-1640 medium supplemented with 10% fetal bovine serum (FBS) and 2% penicillin and streptomycin (PS) at 37°C with 5% CO_2_ in cell culture incubators. Palmitic acid (PA, Cat#P0500) and D-Glucose (Cat#D9434) was purchased in Sigma-Aldrich Co.,Ltd, MO, USA. The detailed grouping conditions are as follows: Ctrl (normal culture medium), PA+HG (100 μM palmitic acid+33.3 mM glucose medium), PA+HG+Dapa 20 μM (100 μM palmitic acid+33.3 mM glucose medium+20 μM dapagliflozin), PA+HG+Dapa 40 μM (100 μM palmitic acid+33.3 mM glucose medium+40μM dapagliflozin).

The proliferation and cell viability of HL-7702 cells were assessed using the Cell Counting Kit-8 (CCK-8) assay (Cat#BMU106, Abbkine Scientific Co.,Ltd, Wuhan). To be precise, HL-7702 cells (10^4^ cells/well) were placed in 96-well plates and then exposed to different concentrations of PA (0, 20, 50, 100, 200, and 300 μmol/L) for a duration of 24 hours. Subsequently, we introduced 10 μL of CCK-8 into each well and added the cells to a 1-hour incubation for 37°C. Finally, we used the enzyme-labelled Instrument to measure the OD value at 450 nm, and the cell viability was calculated according to the manufacturer’s instructions.

### 2.4 Weighted gene co-expression network analysis and differential genes analysis of GEO datasets

WGCNA, a gene co-expression network was established to explore gene-phenotype co-expression with the R software package. (1) Construct a scale-free gene co-expression network using 101 samples and 15384 genes with no missing values and correlation coefficients to create a similarity matrix in GSE13270. (2) Module identification: The topological overlap matrix (TOM) measured the average network connectivity for each gene. According to “minModuleSize” (50) and “mergeCutHeight” (0.25), each module has a separate colour, with the gray module holding unallocated genes. The module feature gene, a first principle component module eigengene (ME), was used to determine the link between modules and phenotype. The highest absolute correlation module was chosen for further study. (3) “GS” and “MM” analysis. Gene significance (GS) was demonstrated the relationship between genes and phenotype. Module membership (MM) is the correlation coefficient, which indicates the relationship between the gene and module.

Meanwhile, the differential genes analysis were constructed using “ggplot2” package in the R software. In the DESeq2 analysis, differentially regulated genes were defined as those with a two-fold change, with an adjusted *P < 0.05*. Subsequently, the selected data were imported into SRplot (http://www.bioinformatics.com.cn/) for visualization.

### 2.5 Analysis of the overlapping genes from dapagliflozin and diabetic related liver injury

First of all, the SwissTargetPrediction and GeneCards databases were used to obtain the genes of Dapagliflozin. As for the genes of diabetic liver injury, “diabetes mellitus” as the keyword was searched in the DisGeNET, GeneCards, OMIM, and TTD databases. The GSE13270 and GSE2899-related genes were merged to remove duplicate genes. Genes related to dapagliflozin and diabetic liver damage were imported into SRPLOT (https://www.bioinformatics.com.cn/) to obtain overlapping genes for visualization. Second, the overlapping genes were imported into the Metascape (https://metascape.org/) website for MCODE, GO and KEGG enrichment analysis. Humans (*Homo sapiens*) were used as the screening criteria for the customized analyses. Subsequently, the pathway enrichment analysis data were imported into the free online bubble chart platform SRPLOT for visualization.

### 2.6 Construction of drug-genes-pathway diagram and screening of hub genes

The top 10 KEGG pathway ranked in gene ratio were selected to perform the drug-genes-pathways diagram. The two files (“network” and “type”) were imported and the hub genes were selected using CytoNCA in Cytoscape 3.9.1. In addition, to further explore the expression of hub genes in diabetic liver injury, the violin plots were performed using “ggpubr” package in R software.

### 2.7 Single-cell sequencing analysis

To further determine in which cell type of the liver the related genes may be significantly expressed, we analysed the data of GSE239612 in GEO datasets. Initially, we performed quality control (200<feature RNA<5000, Percent.mt<20). Subsequently, Uniform Manifold Approximation and Projection (UMAP) and t-distributed Stochastic Neighbor Embedding (tSNE) were performed using R software for dimensionality reduction to obtain more accurate results.

### 2.8 Molecular docking and Molecular dynamics

Initially, we utilised the UniProt and PDB databases (https://www.rcsb.org/) to extract essential target information and get the PDB file for the protein receptor. Subsequently, we employed PyMOL software to exclude water molecules and residues, providing a PDB format file for the macromolecular protein receptor. We employed the TCMSP and PubChem databases to acquire data on active components, resulting in the procurement of a 2D structure file (SDF format) for the ligand. The dataset was subsequently optimised into a 3D structure via Chem3D software, yielding a mol2 format file for the small molecule ligand. Ultimately, we utilised AutoDock program, employing the Grid and Docking modules to execute the docking between the protein and the ligand, and subsequently visualised the molecular docking results using PyMOL software.

Molecular dynamics (MD) simulations were performed using the AMBER 18 software package. Prior to the simulation, energy minimization of the system was carried out using 2500 steps of the steepest descent method followed by 2500 steps of the conjugate gradient method. After energy minimization, the system was gradually heated from 0 K to 298.15 K over 200 ps under a fixed volume and a constant heating rate. Subsequently, an NVT (constant volume and temperature) equilibration was conducted for 500 ps at 298.15 K to ensure uniform solvent distribution within the solvent box. This was followed by a 500 ps NPT (constant pressure and temperature) equilibration simulation. Finally, the two complex systems underwent 100 ns NPT simulations under periodic boundary conditions. During the simulation, a non-bonded interaction cutoff of 10 Å was applied, and long-range electrostatic interactions were computed using the Particle Mesh Ewald (PME) method. The SHAKE algorithm was employed to constrain hydrogen bond lengths, and temperature regulation was performed using the Langevin thermostat with a collision frequency (γ) set to 2 ps^−1^;. The system pressure was maintained at 1 atm, with an integration time step of 2 fs. Trajectories were saved every 10 ps for subsequent analysis.

### 2.9 H&E staining

According to the standard protocols, livers were fixed in 4% paraformaldehyde for 24 h and dehydrated overnight, then the tissues were embedded in embedding agent OCT and frozen sections (5 µm) were prepared at -20°C for H&E staining. Following the manufacturer’s directions, the mice liver slices were stained using the H&E kit (catalog number: G1076, Servicebio, Wuhan, China). All images were obtained using the microscope (BX53; Olympus Corporation, Tokyo, Japan).

### 2.10 Western blot

The liver tissues were processed to extract total proteins for Western blot using a RIPA buffer (Cat#P0013B, Beyotime Biotechnology, Shanghai). The lysis buffer consisted of 1% Phenylmethanesulfonyl fluoride (PMSF) (Cat# G2008, Servicebio, Wuhan, China) and 1% Phosphoprotease inhibitor (Cat# G2007, Servicebio, Wuhan, China). The BCA protein detection kit (Cat#MA0082, Meilunbio, Dalian, China) was employed for the quantification of these proteins. The Western- blot technique was performed using the previously reported methodology (23). In summary, protein samples ranging from 20 to 40 μg were analysed using a 10% to 12% (w/v) SDS-PAGE gel (Cat#PG212/PG213; Epizyme, shanghai). The proteins that had been isolated were subjected to electroblotting and subsequently deposited onto a polyvinylidene difluoride (PVDF) membrane. The membrane was blocked using TBST solution that consisted of 5% nonfat milk or bovine serum albumin. Subsequently, the membranes were subjected to an overnight at 4°C with primary antibodies. And its was subjected to incubation with secondary antibodies (Cat#L3032/L3012, 1:10000, Signalway Antibody, Greenbelt, MD, USA) at ambient temperature. The visualisation of protein blots was achieved by the utilisation of an ECL system and the Image Lab detection system, manufactured by BioRad in Hercules, CA. GAPDH, β-actin, or α-tubulin were employed to normalise the protein bands and examine them using Image J.

### 2.11 qPCR analysis

The total RNA of liver has been obtained using RNAeasy™ Animal RNA Isolation Kit with Spin Column (Cat#R0026, Beyotime Biotechnology, Shanghai). The RNA content was assessed using an ultra-microspectrophotomete. Then, complementary DNA (cDNA) was obtained from the RNA through the process of reverse transcription, utilizing a HiScript II Q RT SuperMix (Cat#R222-01; Epizyme, shanghai). Afterwards, SYBR-Green method was used for real-time quantitative cDNA amplification (Cat#Q711-02; Epizyme, shanghai). Finally, the relative mRNA levels have been determined using the 2^−ΔΔCT^ method and standardized against GAPDH. The primer sequences of the genes for qPCR are shown in **Table 1**.

**Table 1:** Primers sequences.

Gene		Primer sequence (5′-3′)
*Bcl2*	Forward	CTTCTCTCGTCGCTACCGTC
	Reverse	CAATCCTCCCCCAGTTCACC
*Bax*	Forward	ACCAAGAAGCTGAGCGAGTGTC
	Reverse	TGTCCACGGCGGCAATCATC
*Casp3*	Forward	GGCTGACTTCCTGTATGCTTACTC
	Reverse	CGACCCGTCCTTTGAATTTCTCC
*Ikbkb*	Forward	CGAAGACTTGAATGGAACGGTGAAG
	Reverse	GCCACTTCTCCAGTCGCTCAG
*Nfkb1*	Forward	ATCATCCACCTCCACGCTCAG
	Reverse	TCCTCTACTACATCTTCCTGCTTGG
*Il6*	Forward	CTTCTTGGGACTGATGCTGGTGAC
	Reverse	AGGTCTGTTGGGAGTGGTATCCTC
*Il1β*	Forward	TGCTGGTGTGTGACGTTCCC
	Reverse	ATGGGTCCGACAGCACGAG
*Il18*	Forward	AAATGACCAAGTTCTCTTCGTTGAC
	Reverse	CACAGCCAGTCCTCTTACTTCAC
*Gapdh*	Forward	AAGTTCAACGGCACAGTCAAGG
	Reverse	GACATACTCAGCACCAGCATCAC

### 2.12 Statistical analysis

Statistical analysis was conducted using the GraphPad Prism (version 9.00). The experiments were repeated at least three times, and the data are expressed as the mean ± standard deviation (SD). One-way ANOVA and Tukey test were used for comparison among multiple groups. *P<0.05* was considered to indicate statistical significance.

## 3 Results

### 3.1 Dapagliflozin protects against diabetic liver injury by attenuating lipid accumulation

A flowchart of the feeding and harvesting of the mice was shown (**Figure 1A**). As shown in **Figure 1B**, the fasting blood glucose levels were higher (>10 mmol·L^-1^) in the DM group, which was persisted at this elevated level during the subsequent observation period. Obviously, H&E staining of liver tissue clearly demonstrated that dapagliflozin could alleviate hepatocyte steatosis, and the formation of rounded lipid vacuoles (**Figure 1C**). Moreover, the measurement of TG, T-CHO, LDL-C, and HDL-C concentrations was proper to estimate qualitative change of lipid accumulation (24). As shown in **Figures 1D–G**, dapagliflozin also decreased the TG, T-CHO, and LDL-C content and increased the HDL-C content in liver tissue of mice caused by diabetes (DM group). These findings indicated that dapagliflozin could improve the lipid accumulation in the diabetic liver injury mice.

### 3.2 Dapagliflozin protects against diabetic liver injury by improving oxidative stress

**Figure 2:**
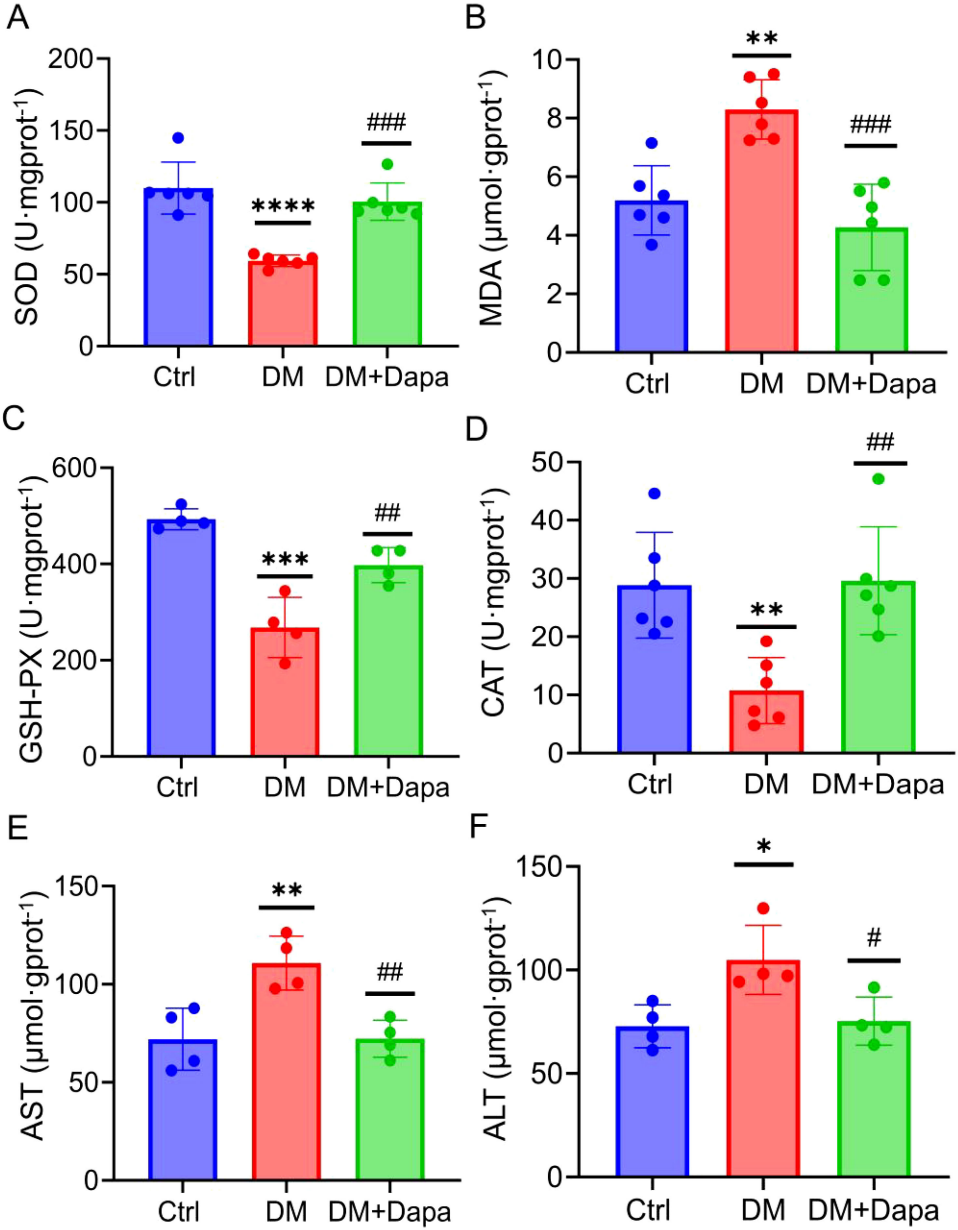
Dapagliflozin protects against diabetic liver injury by improving oxidative stress. **(A–D)** Dapagliflozin alleviated oxidative stress on SOD, MDA, GSH-PX, and CAT activity in the DM group (n≥4). **(E, F)** Aspartate aminotransferase (AST) activity and alanine aminotransferase (ALT) activity (n=4). SOD, Superoxide dismutase; MDA, Malondialdehyde; GSH-PX, Glutathione peroxidase; CAT, Catalas; AST, Aspartate transaminase; ALT, alanine aminotransferase. ^*^P < 0.05, ^**^P < 0.01, ^***^ *P < 0.001*, ^****^ *P < 0.0001* vs. the Ctr group; ^#^ *P < 0.05*, ^##^ *P < 0.01*, ^###^ *P < 0.001* vs the DM group.

Oxidative stress is recognized as a key factor in the development of liver injury, especially within the framework of the ‘multiple hits’ hypothesis. As expected, the content of SOD, GSH-PX, and CAT was markedly increased in comparison to the DM group, whereas the levels of MDA was reduced when the dapagliflozin were feed (**Figures 2A–D**). Furthermore, dapagliflozin could significantly reduce the AST and ALT content in the liver tissue after dapagliflozin treatment (**Figures 2E, F**). Taken together, dapagliflozin could ameliorate diabetic liver injury by attenuating oxidative stress levels.

### 3.3 Dapagliflozin protects against diabetic liver injury by inhibiting liver inflammatory and apoptosis proteins

**Figure 3:**
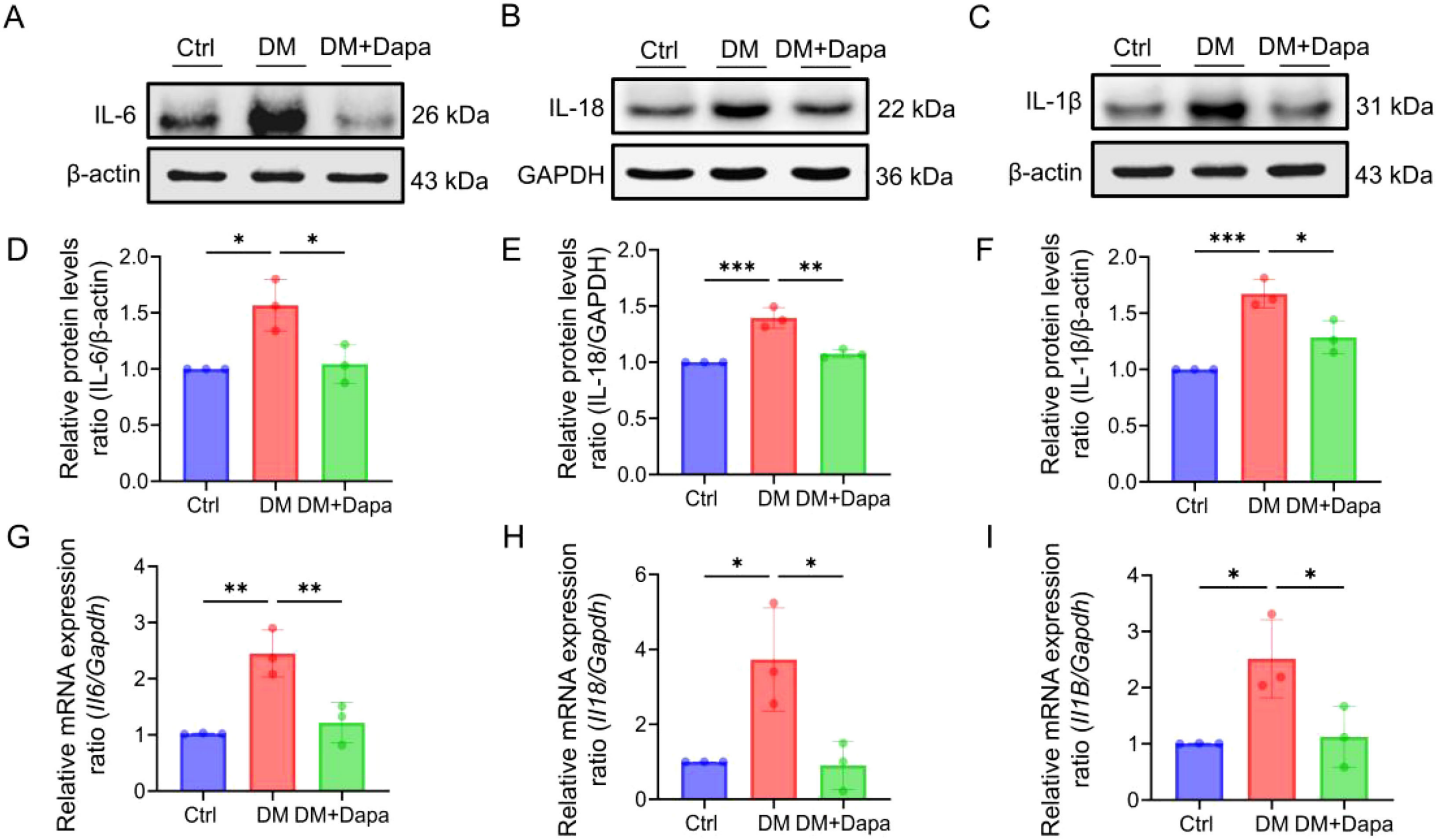
Dapagliflozin could improve liver cells inflammatory response. **(A–I)** The proteins level and mRNA expression in IL-6 (*Il6*), IL-18 (*Il18*) and IL-1β (*Il1β*) was shown, n = 3. ^*^P < 0.05, ^**^P < 0.01, ^***^ *P < 0.001*.

**Figure 4:**
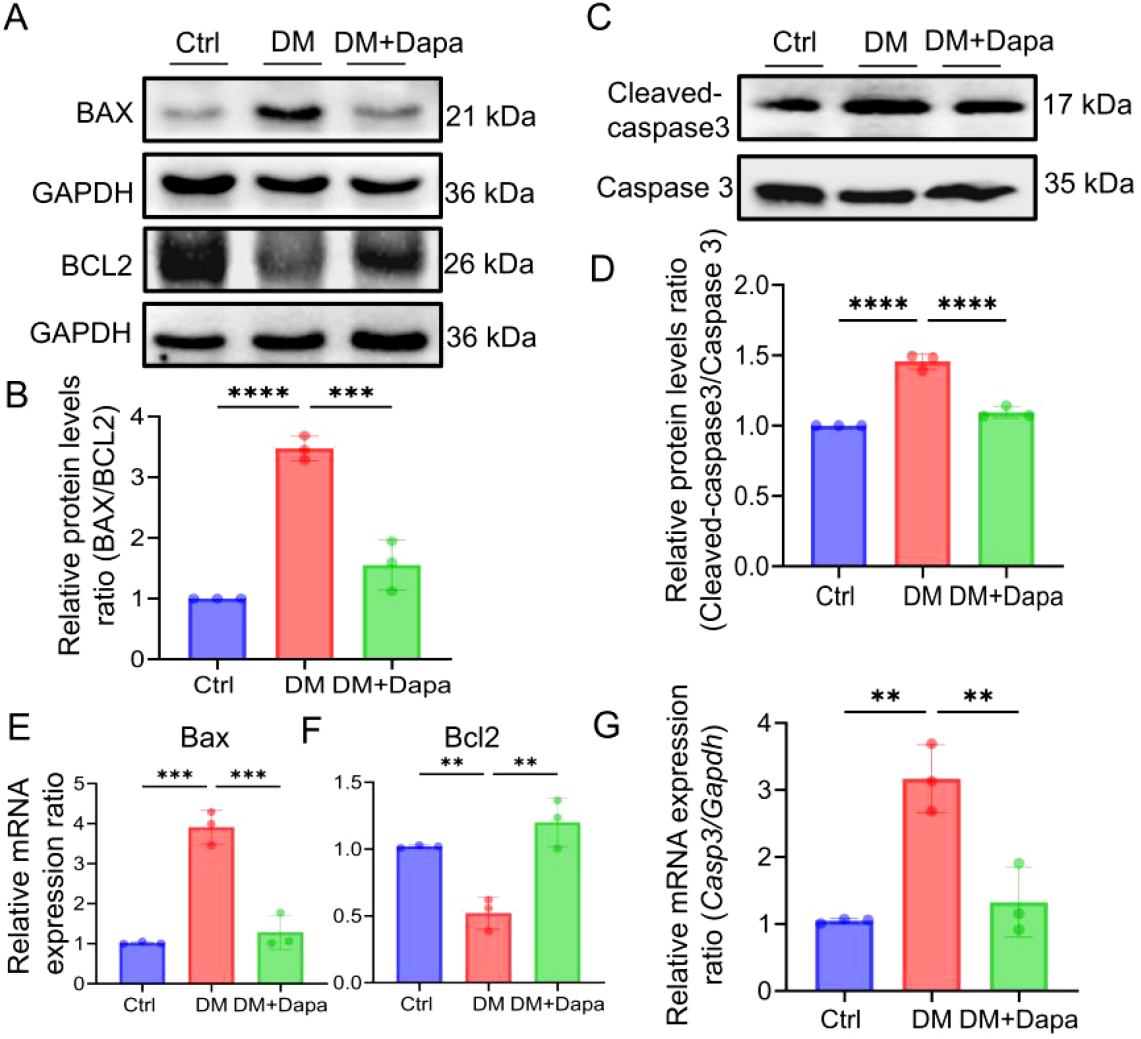
Dapagliflozin could improve liver cell apoptosis. **(A-G)** The proteins level and mRNA expression in BAX (*Bax*), BCL2 (*Bcl2*) and Cleaved-caspase3/Caspase3 (*Casp3*) were detected, n = 3. ^**^P < 0.01, ^***^ P < 0.001, ^****^P < 0.0001.

Given the growing research, the activation of inflammatory pathways is a consequence of the substantial hepatic metabolic stress that is generated by excessive lipid accumulation. The inflammatory protein levels of IL-6, IL-1β, and IL-18 were significantly decreased in the treatment of dapagliflozin (**Figure 3**). It was worth noting that the changes in the mRNA expression of *Il6*, *Il1β*, and *Il18* (**Figures 3G–I**) were in line with the corresponding variations in protein levels (**Figures 3A–F**), respectively. Furthermore, dapagliflozin treatment could decrease the expression of apoptosis-related proteins including Cleaved-caspase3/Caspase3 and BAX/BCL2 (**Figures 4A–D**), which was consistent with the decrease in the mRNA expression of apoptosis-related genes (**Figures 4E–G**).

Collectively, we determined that dapagliflozin alleviated diabetic liver injury through inhibiting the expression of interleukin-related inflammatory and apoptosis proteins.

### 3.4 Identifying the overlapping of genes between dapagliflozin and diabetic liver injury

To further explore which pathways or genes were involved in the improvement of diabetic liver injury by dapagliflozin. The datasets of GSE2899 and GSE13270 from the GEO database were further conducted in order to identify genes associated with diabetic liver injury. Originally, the data in GSE2899 were mined and presented in **Supplementary Figures S1A, B**, a total of 61 differential expression genes were examined, and the top 10 genes of up-regulated and down-regulated were displayed in the cluster heatmap, respectively.

**Figure 5:**
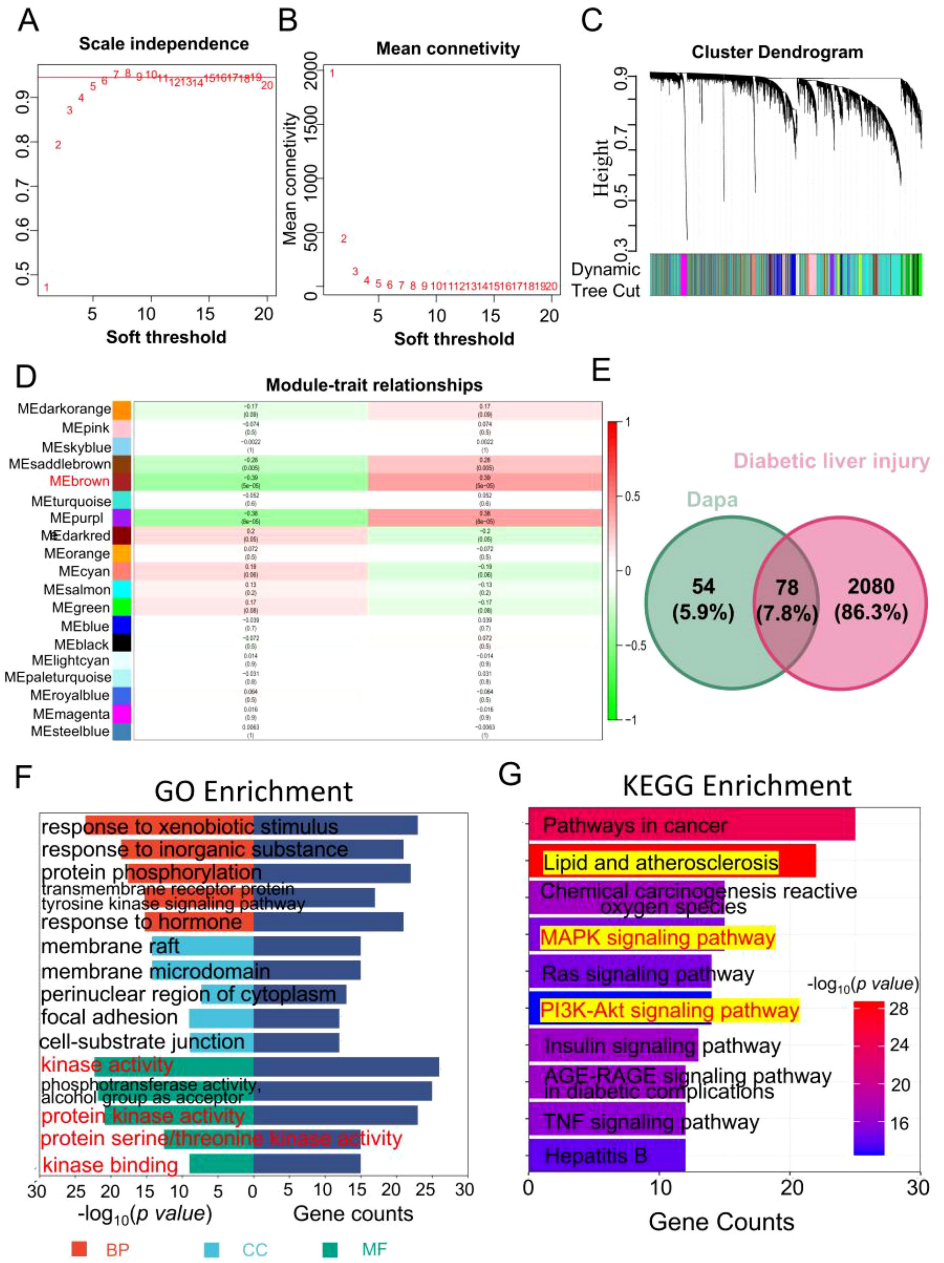
Identifying the overlapping of genes between dapagliflozin and diabetic liver injury. **(A, B)** The soft threshold is obtained and shows the mean connectivity values at different soft threshold value. **(C)** Dendrogram of all differentially expressed genes clustered. **(D)** 19 different gene modules were obtained. Each cell contains the different modules and clinical traits correlation and p-value. **(E)** The Venn diagram of dapagliflozin (132 related genes of dapagliflozin were obtained in the Genecards (37) and SwissTargetPrediction (100) databases, the green circle) and diabetic liver injury (the red circle). **(F)** The GO enrichment analysis including biological process (red), cellular component (blue), and molecular function (green) was performed in the overlapping genes. **(G)** The top 10 KEGG enrichment pathway was shown. The range of colour represents the p value in each pathway, and the number of genes in each pathway was shown according the length of the bar.

Subsequently, the hub modules and genes were obtained using WGCNA within GSE13270. Initially, when, the scale independence metric achieved a value of 0.958 and the soft threshold was 6, while the average connection value reached 32.425 (**Figures 5A, B**). These results indicated the establishment of a scale-free gene network. Afterwards, a total of 19 modules were found by the utilization of average hierarchical clustering and dynamic tree clipping, as depicted in **Figure 5C**. The MEbrown module exhibited a significant down-regulation in diabetic liver injury samples, as indicated by the correlation coefficient (r=-0.39, p=5e-5) in **Figure 5D**. Additionally, the analysis of gene significance (GS) and module membership (MM) revealed a substantial correlation between the eigengenes of the MEbrown module (cor=0.41, p=1.7e-67, **Supplementary Figure S1C**), indicating a strong association between eigengenes and their respective modules. The analysis presented in **Supplementary Figure S1D** demonstrated a strong association between the eigengenes of the MEbrown module and diabetic liver injury. 57 genes were selected in the MEbrown module (|GS|>0.3 and |MM|>0.6).

Finally, a total of 2051 genes associated with diabetes mellitus were found following the process of combining and eliminating duplicates within the GeneCards (2043), OMIM (20), DisGeNET (7), and TTD (7) databases. And 2158 diabetic liver injury genes were obtained using the GEO datasets and databases.

As shown in **Figure 5E**, 78 overlapping genes from dapagliflozin (132) and diabetic liver injury (2158) were obtained. At the same time, the pathway analysis (GO and KEGG) were conducted based on the overlapping genes. As shown in **Figure 5F**, biological process (BP) terms related to response to xenobiotic stimulus, response to inorganic substance, protein phosphorylation, cellular component (CC), response to membrane raft, membrane microdomain, perinuclear region of cytoplasm, and molecular function (MF) terms included kinase activity, protein kinase binding, and protein serine/threonine kinase activity. The top 10 KEGG pathways were collected in **Figure 5G**. Notably, the Lipid and atherosclerosis was the potential pathways for further investigation.

### 3.5 The hub genes of dapagliflozin attenuates diabetic liver injury were selected

**Figure 6:**
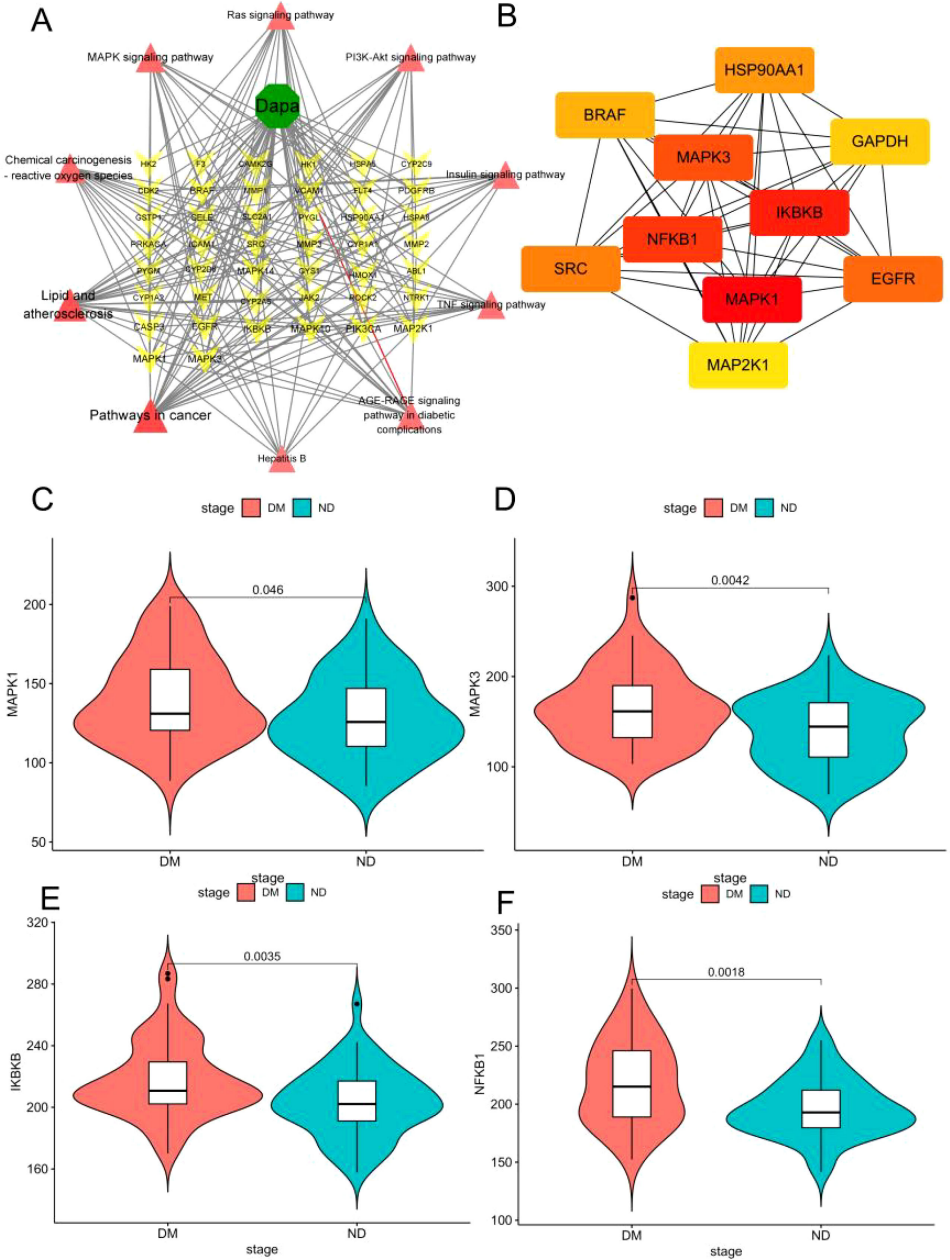
The hub genes of dapagliflozin attenuates diabetic liver injury were selected. **(A)** Dapa-targets-pathways diagram. The red triangle represents the top 10 pathways of KEGG enrichment, the yellow triangular pyramid represents genes, and the green octagon represents dapagliflozin. **(B)** The Cytohubba analysis of the top 10 genes in Cytoscape 3.9.1 was shown. The range of colour represents the importance of overlapping genes. **(C–F)** The violin plot of *Mapk3, Mapk1, Ikbkb, and Nfkb1* in diabetic liver injury and non-diabetic groups.

Based on the results shown in **Figure 5G**, it was necessary to select the hub genes from genes in the top 10 pathways of KEGG enrichment. First, the dapagliflozin-diabetic liver injury-pathways diagram that consisted of the top 10 pathways and 38 relevant genes was conducted in **Figure 6A**. *Mapk1, Mapk3, Ikbkb*, and *Nfkb1* were hub genes in the Cytohubba analysis (**Figure 6B**). Furthermore, the violin plots was conducted to valid the hub genes expression in diabetic liver injury group based on GSE13270. As shown in **Figures 6C–F**, *Mapk1, Mapk3, Ikbkb*, and *Nfkb1* were expressed at higher levels in the diabetic liver injury group. Notably, ERK (*Mapk1, Mapk3*), IKBKB *(Ikbkb)*, and NF-κB (*Nfkb1*) were included in Lipid and atherosclerosis (**Supplementary Figure S1**). Therefore, it indicated that ERK2 (*Mapk1*), ERK1 (*Mapk3*), IKBKB (*Ikbkb*), and NF-κB (*Nfkb1*) were the potential targets of dagliflozin treating diabetic liver injury, which merited a further study.

### 3.6 Molecular docking and molecular dynamics to verify the binding ability of dapagliflozin and hub proteins

**Figure 7:**
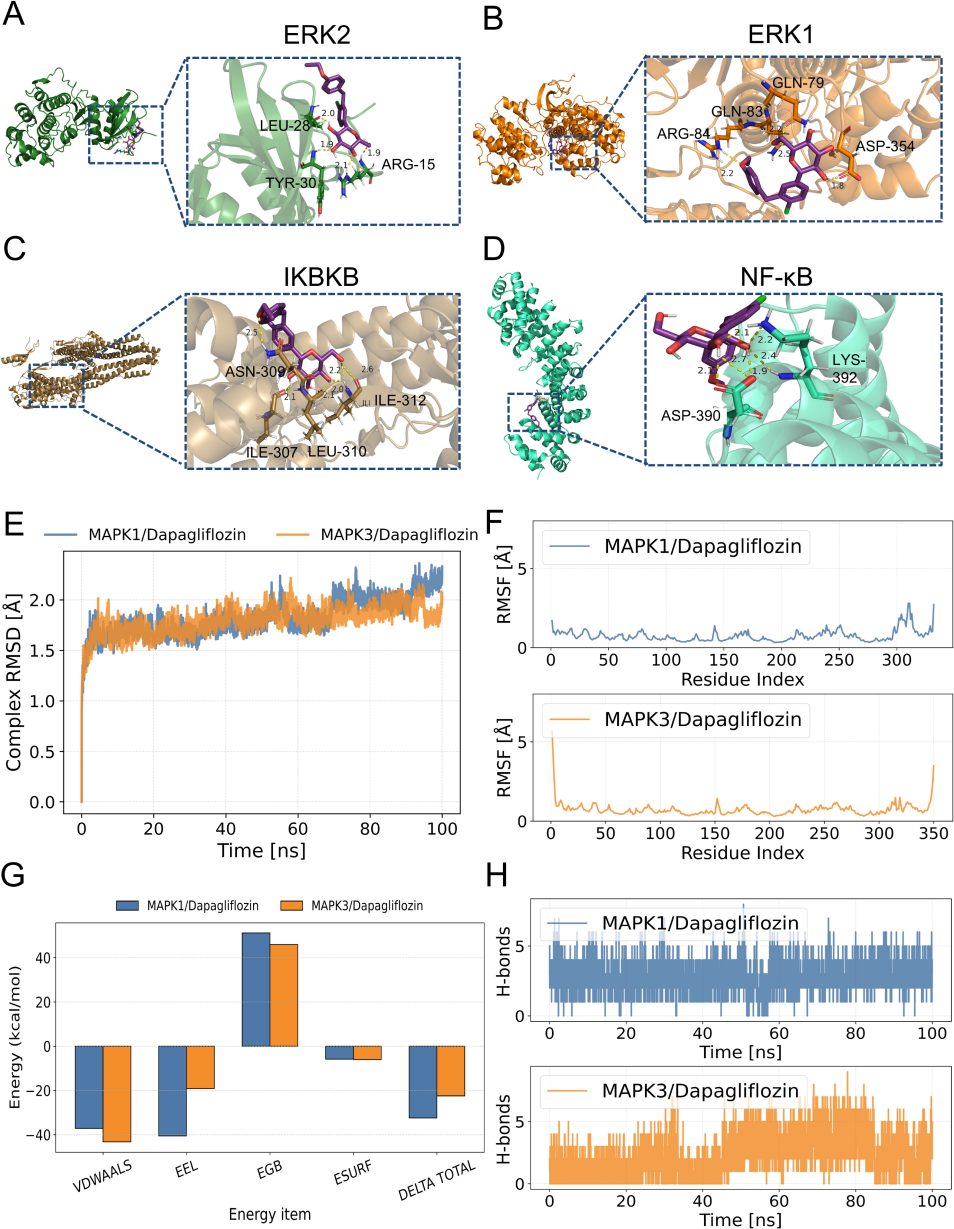
Molecular docking to verify the binding ability of dapagliflozin and hub proteins. The magenta molecule represents a ligand (dapagliflozin), the yellow dotted line represents hydrogen bonds between the ligand and receptor, and the forest, orange, brown, and greencyan represent fragments of ERK2, ERK1, IKBKB and NF-κB, respectively. **(A)** dapagliflozin and ERK2. **(B)** dapagliflozin and ERK1. **(C)** dapagliflozin and IKBKB. **(D)** dapagliflozin and NF-κB. **(E)** Changes of root mean square deviation (RMSD) of the complex over time during molecular dynamics simulation. **(F)** Root mean square fluctuation (RMSF) calculated based on molecular dynamics simulation trajectories. **(G)** MM-GBSA binding energy and energy decomposition. **(H)** Changes in the number of hydrogen bonds between small molecules and proteins during molecular dynamics simulation.

The relative affinities of dapagliflozin and the hub proteins were determined via molecular docking. In particular, two hydrogen bonds with ARG-15 and one with TYR-30 and LEU-28 in ERK2 were formed with dapagliflozin (**Figure 7A**). Similarly, dapagliflozin could form one hydrogen bond with GLN-83, ARG-84, GLN-79, and ASP-354 in ERK1 (**Figure 7B**). Meanwhile, **Figure 7C** showed that dapagliflozin formed three hydrogen bonds with ILE-312 and one hydrogen bonds with ILE-307, ASN-309, and LEU-310 in IKBKB. Finally, as for NF-κB, it was shown that dapagliflozin formed three hydrogen bonds with ASP-390 and LYS-392 (**Figure 7D**). It was commonly believed that the ligand-receptor binding energy values were less than -5 kcal·mol^-1^, and the lower energy conformation was more stable (25). Notably, dapagliflozin and ERK1 had the lowest binding energy (-8.17kcal·mol^-1^), demonstrating the highest affinity between them (**Table 2**).

**Table 2:** The binding energy of dapagliflozin (Dapa) and the hub proteins.

Drug	Protein	Protein (PUB ID)	Affinity(kcal·mol^-1^)
Dapa	ERK2	6slg	-8.05
	ERK1	6ges	-8.17
	IKBKB	5tqy	-5.44
	NF-κB	7let	-6.76

To further determine the binding affinity of ERK1 and ERK2 with dapagliflozin, molecular dynamics (MD) simulations were performed. As shown in **Figure 7E**, both complexes exhibited good structural stability throughout the 100 ns simulation, without significant conformational collapse. Among them, the MAPK3/Dapagliflozin complex showed slightly better performance in terms of RMSD values and fluctuation range, suggesting a potentially higher binding stability. Additionally, except for local regions of the protein, the RMSF values of the protein remained within 2 Å, indicating a high degree of structural rigidity, which may be attributed to the binding of the small molecule dapagliflozin (**Figure 7F**). Furthermore, based on the MD simulation trajectories, the binding free energy was calculated using the MM-GBSA method. The binding free energies of the MAPK1/Dapagliflozin and MAPK3/Dapagliflozin complexes were -32.44 ± 3.3 kcal/mol and -22.46 ± 3.75 kcal/mol, respectively. These results suggest favourable binding interactions in both cases (**Figure 7G**). Finally, we monitored the number of hydrogen bonds formed between the ligand and the protein during the 100 ns simulation (**Figure 7H**). The results showed that the MAPK1 complex maintained relatively stable and frequently fluctuating hydrogen bonds throughout the simulation, with numbers ranging from 1 to 6, exhibiting significant fluctuations but an overall uniform distribution. In contrast, the MAPK3 complex initially showed fewer hydrogen bonds within the first 30 ns, followed by a gradual increase, stabilizing between 2 and 6 hydrogen bonds in the later stages, suggesting an increasing trend in hydrogen bond formation.

As a result, it was clear from our results that dapagliflozin had a better affinity and more stable conformation of ERK1 compared to other proteins.

### 3.7 Dapagliflozin regulates diabetic liver injury by ERK/IKKβ/NF-κB signalling pathway *in vivo* and *in vitro*

**Figure 8:**
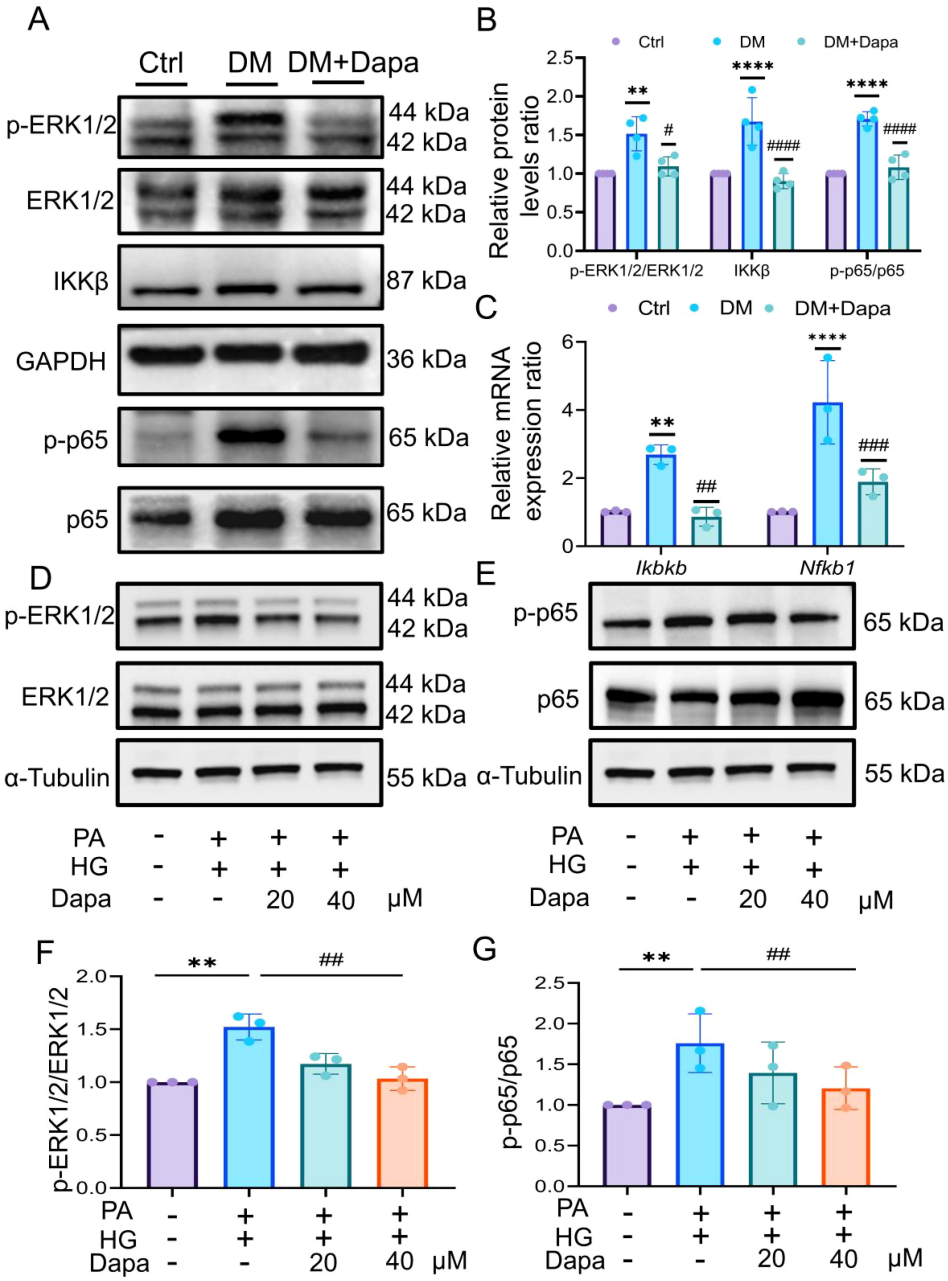
Dapagliflozin could inhibit the ERK/IKKβ/NF-κB signalling pathway to improve diabetic liver injury. **(A, B)** The proteins level in ERK1/2, IKKβ, and p65 was shown, n = 4. **(C)** The expression of *Ikbkb* and *Nfkb1* was analysed by qPCR. **(D–G)** The proteins level in ERK1/2 and NF-κB p65 was explored by western blot, n = 3. qPCR, Realtime fluorescence quantitative; CCK-8, Cell Counting Kit-8; PA, palmitic acid; HG, high glucose. ^**^P < 0.01, ^****^ *P < 0.0001* vs.Ctr group; ^#^ *P < 0.05*, ^##^ *P < 0.01*, ^###^ *P < 0.001*, ^####^ *P < 0.0001* vs DM group.

To confirm whether dapagliflozin ameliorates diabetic liver injury through specific target proteins, their levels were measured in experiments. First, the phosphorylation of ERK1/2 (THR-202/TYR-204) and NF-κB (P65) increased in the DM group, and the protein levels significantly decreased after dapagliflozin administration. In addition, the protein levels of IKKβ was decreased following treatment with dapagliflozin. (**Figures 8A, B**). As shown in **Figure 8C**, dapagliflozin treatment could down-regulated the mRNA expression of *Ikbkb* and *Nfkb1* in DM group, consistent with their protein levels.

Moreover, we verified these results in HL-7702 cells in vitro experiments. First, we tested the protein level of SGLT2. The results showed that the induction of PA and HG had no significant effect on the expression of SGLT2 (**Supplementary Figure S5C**). Furthermore, the phosphorylation level of ERK1/2 and NF-κB were decreased after treatment with 20μM and 40μM dapagliflozin (**Figures 8D–G**), but 40μM of which treatment was statistically significant (*P<0.05*). To further verify the upstream-downstream relationship between ERK and NF-κB, the ERK inhibitor SCH772984 was introduced. The results showed that treatment with PA and HG significantly increased the phosphorylation level of NF-κB, while co-treatment with SCH772984 markedly suppressed NF-κB phosphorylation (**Supplementary Figure S5D**).

Therefore, the results indicated that dapagliflozin might inhibit the ERK/IKKβ/NF-κB signalling pathway, thereby improving diabetic liver injury.

## 4 Discussion

**Figure 9:**
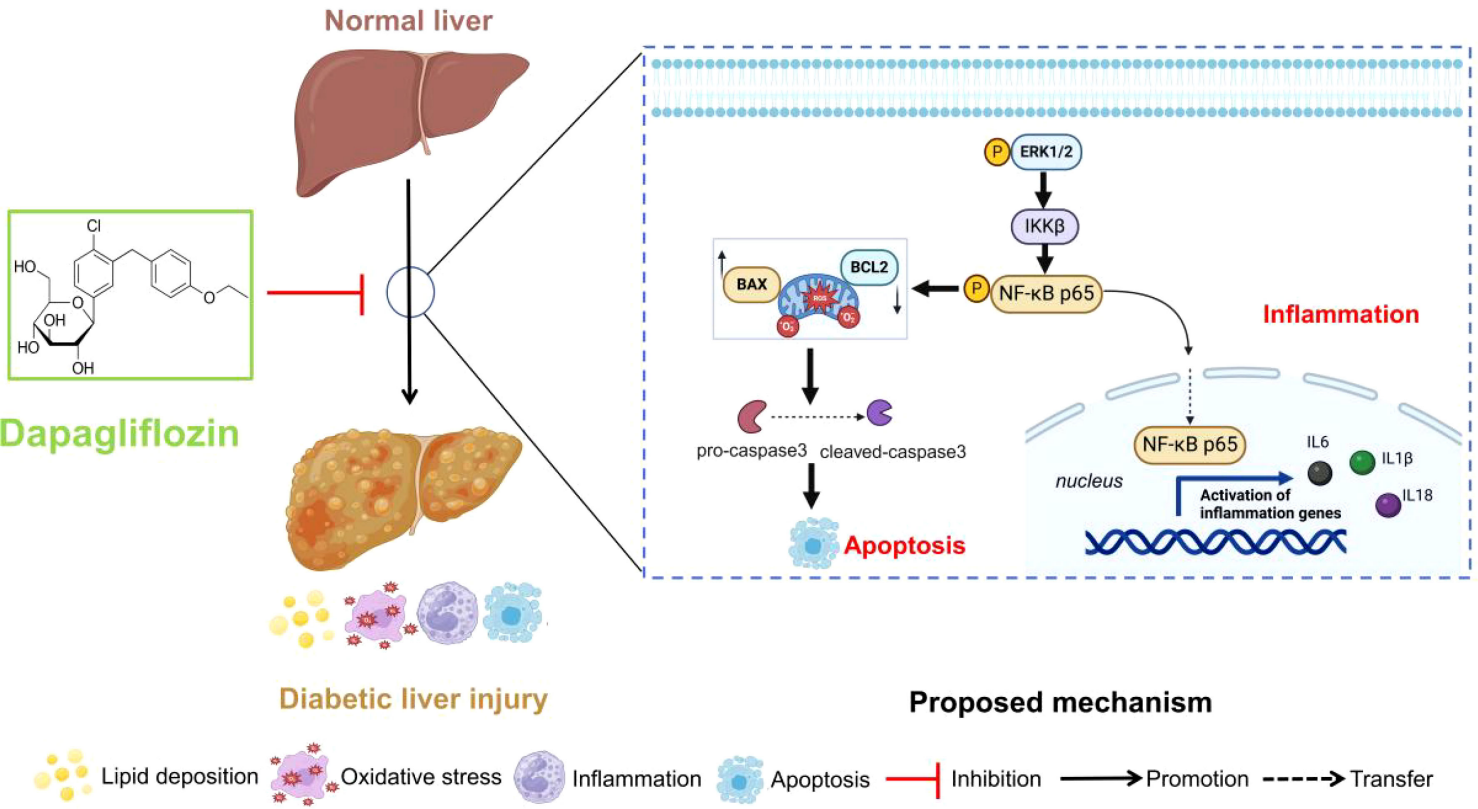
Proposed mechanism of dapagliflozin in the regulation of diabetic liver injury.

In this study, dapagliflozin could ameliorate the diabetic liver injury by reducing the lipid deposition, oxidative stress levels and inflammatory and apoptosis-related proteins and mRNA expression. Furthermore, *Mapk3*, *Mapk1*, *Ikbkb* and *Nfkb1* were identified to treat diabetic liver injury by dapagliflozin in GSE13270 and pharmacology databases. Subsequently, the related proteins and genes were detected *in vivo* and *in vitro* experiment verification. Collectively, it concluded that dapagliflozin could regulate diabetic liver injury via the ERK/IKKβ/NF-κB signalling pathway. The proposed schematic of dapagliflozin in the regulation of diabetic liver injury was shown in **Figure 9**.

Dapagliflozin could inhibit SGLT2 in the proximal renal tubule, thereby reducing sodium and glucose re-absorption. Studies have shown that dapagliflozin activates the AMPK pathway and inhibits pro-inflammatory cytokines to improve and reduce streptozotocin-induced renal injury in diabetic mice (26). In addition, dapagliflozin could improve ischemia/reperfusion damage in obese insulin-resistant rats by reducing myocardial infarct size and improving cardiac mitochondrial function (27). Moreover, it is essential to highlight that dapagliflozin has the capacity to enhance the expression of FXR/SHP and regulate the AMPK/mTOR pathway, hence resulting in a decrease in hepatic lipogenesis in db/db mice (28, 29). In our study, the triglycerides (TG), total cholesterol (T-CHO), high-density lipoprotein cholesterol (HDL-C), and low-density lipoprotein cholesterol (LDL-C) concentrations in the liver tissue of diabetic mice were affected after dapagliflozin intervention., which was alignment with the findings of H&E staining. The results showed that diabetic liver injury induced by a high-fat diet combined with STZ not only produced mild steatosis, but also focused on hepatocyte inflammation and apoptosis. Therefore, this study determined a new mechanism by which dapagliflozin could improve diabetic liver injury.

Previous investigation has demonstrated a correlation between the prevalence of the majority of liver diseases and oxidative stress (30). MDA, SOD, GSH, CAT, AST, and ALT levels were used to monitor STZ-induced liver oxidative damage. MDA is a cell oxidative metabolite, which can reflect the degree of lipid peroxide damage, the increase in MDA content is considered a typical feature of liver injury (31, 32). Hydrogen peroxide is an important reactive oxygen species that can damage the liver by promoting oxidation. Apparently, it protects tissues and cells in diabetic livers from oxidative injury (33). GSH and SOD are both essential scavengers of endogenous oxygen free radicals. SOD, an endogenous peroxidase, regulates the levels of hydrogen peroxide and superoxide generated by cells and safeguards cellular structures against oxidative stress-induced damage. GSH has physiological functions such as free radical scavenging, antioxidant and detoxification, and ROS/GSH balance damage, which can lead to oxidation and chemical modification of biological macromolecules (34, 35). In addition, studies have shown that AST and ALT were considered important indicators for evaluating diabetic liver injury (36). The results of this study showed that dapagliflozin could reduce the content of MDA in the liver and increase the content of SOD, GSH-PX, and CAT, indicating that dapagliflozin can improve liver oxidative stress and reduce liver injury in diabetic mice.

Network pharmacology was initially proposed by Li et al. as an important component of bioinformatics, and its significance in compound acquisition has gained increasing attention (37). However, numerous studies have indicated that the targets and pathways are often redundant with limited biological significance, which might be due to the use of the same binary algorithms for screening disease targets. Weighted Gene Co-expression Network Analysis (WGCNA), based on scale-free topology criteria, is closer to biologically motivated standards (38). WGCNA can cluster a large number of disease-related genes into co-expression modules and validate gene modules associated with the clinical characteristics of samples based on gene expression pattern coefficients. This enables the use of more complex and biologically relevant algorithms for gene screening. Mechanically, 78 overlapping genes of dapagliflozin and diabetic liver injury were obtained. Notably, *Mapk3, Mapk1, Ikbkb*, and *Nfkb1* as the hub genes involved in dapagliflozin attenuating diabetic liver injury were identified, and dapagliflozin exhibited the better affinity with these proteins in molecular docking.

The extracellular signal-regulated kinase 1/2 (ERK1/2) cascade is a central signalling pathway, which play an important role in mitogen-activated protein kinase (MAPK) signalling transduction pathways (39). It is involved in mitotic signalling to regulate the cell cycle and affects cell division and apoptosis (40, 41). Notably, previous research has indicated that MAPKinase substrates activate the NF-κB signalling pathway (42), which corresponds to the research results of this article. Pan X et al. (43) reported that dapagliflozin could inhibit ERK1/2 phosphorylation and ameliorate fibrosis in the diabetic kidneys. As shown in the results of **Supplementary Figure S1**, the ERK1/2 and NF-κB cascade pathway is a subpathway of lipid and atherosclerosis. Interestingly, Chou AH et al. (44) found that curcumin protects against APAP-induced liver damage by inhibiting the ERK1/2 and NF-κB proteins. However, there is few literature has explored the relationship between dapagliflozin and ERK/NF-κB in the treatment of liver-related diseases. In our result, it indicated that the ERK1/2, BAX/BCL2 and cleaved-caspase3/caspase3 protein expression were inhibited in treatment of dapagliflozin (**Supplementary Figure S5B**). Of notes, *Bax* had significant expression in Kupffer cells, whereas *Bcl2* was effectively down-regulated in B cells, as determined by single-cell sequencing (**Supplementary Figure S4**). Apoptosis of Kupffer cells is the pathogenesis of liver damage caused by various diseases. Studies have shown that Kupffer cells are related to hepatocyte apoptosis, inflammation, and fibrosis processes during liver fibrosis, and promoting M2-induced M1 Kupffer cell apoptosis may be a relevant strategy to limit alcoholic fatty liver disease (44, 45). Therefore, dapagliflozin can inhibit the ERK1/2 and may inhibit the apoptosis of Kupffer cells and B cells in the liver, which is closely related to the improvement of diabetic liver injury.

The nuclear factor kappa-B (NF-κB) pathway has been widely recognized as a paradigmatic proinflammatory signalling pathway (46). Phosphorylated NF-κB is activated by IKKβ and then translocates to the nucleus, where it promotes promoting the transcription of inflammatory factors (47). The inflammatory response following liver injury has received widespread attention. Studies have shown that inflammatory immune cells after liver injury mainly include liver-resident macrophages, neutrophil cells, T cells and infiltrating monocytes (48). As shown in **Supplementary Figure S3**: *Nfkb1* was highly expressed in diabetic liver T cells, while *Mapk1* were higher expressed in neutrophil cells. Hu et al (49) believed that the MEK1/2/ERK1/2 and NF-κB were cascade pathways and activate the NF-κB protein level could promote the expression of IL-6 in osteoblasts. Therefore, the ERK1/2/NF-κB pathway may be closely related to the inflammatory response after liver injury. In addition, the relationship between IKKβ/NF-κB and apoptosis has been widely reported. The research considered that natural flavonoids inhibits the IKKβ/NF-κB pathway and inflammatory factors and has a significant effect on improving insulin resistance and pancreatic β cells apoptosis (50, 51). Meanwhile, our results showed that after treatment with dapagliflozin, the expression of IKKβ, p-p65 and inflammatory factors (IL-6, IL-18, IL-1β, and TNF-α) in liver tissue was decreased (**Supplementary Figure S5A**), which preliminarily showed dapagliflozin could inhibit the expression of inflammatory and apoptosis factors by suppressing the ERK/IKKβ/NF-κB signalling pathway, thereby inhibiting the development of inflammation in liver tissue.

In contrast to previous research, this work employs single-cell data analysis to preliminary predict which individual cell types dapagliflozin may affect in the diabetic liver injury. However, other datasets need to be analysed, and the cell types were verified through further study. Additionally, we will validate the cytotoxic effects of dapagliflozin on different liver cells induced by PA and HG in *in vitro* experiments. Regarding the underlying mechanism, we will further knock down ERK1/2 to provide stronger theoretical support for the role of dapagliflozin in alleviating liver injury through the ERK/IKKβ/NF-κB signalling pathway.

In conclusion, dapagliflozin reduces lipid accumulation and oxidative stress, while also alleviating the inflammatory response and decreasing liver cell apoptosis by inhibiting the ERK/IKKβ/NF-κB signalling pathway in mice, as demonstrated by bioinformatics analysis combined with *in vivo* and *in vitro* experiments.

## Data Availability

The original contributions presented in the study are publicly available. This data can be found here: https://www.jianguoyun.com/p/DenYyZcQ44qxDRienYIG.

## Supplementary material

Supplementary Figure 1
**(A)** The volcano plot were display after the analysis of GSE2899, the blue and red dots represents the down-regulated and up-regulated genes, respectively. **(B)** The heatmap of top 10 down-regulated and up-regulated genes were conducted. **(C)** GS and MM analysis were constructed in MEbrown module. **(D)** The visualization of the eigengene network represents the relationship among the modules (MEbrown) and traits (diabetic liver injury). **(E, F)** The MCODE of overlapping genes of dapagliflozin and diabetic liver injury.The blue and red network represents the MCODE1 and MCODE2 network, respectively. The top 3 KEGG pathways of MCODE1 and MCODE2 were enriched in **(F)**.

Supplementary Figure 2 The Lipid and atherosclerosis pathway. (Source: https://www.genome.jp/entry/map05417). The ERK2 (Mapk1), ERK1 (*Mapk3*), IKBKB (*Ikbkb*), and NF-κB (Nfkb1) were marked in red.

Supplementary Figure 3 Single cell sequencing analysis in GSE239612. **(A, B)** Initially, we performed quality control (200<feature RNA<5000, Percent.mt<20). **(C, D)** Uniform Manifold Approximation and Projection (UMAP) and t-distributed Stochastic Neighbor Embedding (tSNE) were perform in R software to dimensionality reduction to obtain more accurate results As shown in **Figures 4C, D**, it was concluded that Macrophage cells, Dendritic cells and Kupffer cells were increased in the DM group, but B cells and Endothelial cells were significantly decreased in the DM group, compared with the ND group in UMAP and tSNE analysis.

Supplementary Figure 4 Expression of the genes in different cell types in the liver. 10 common cell types in the diabetic liver injury were analysed, with different colours representing different cell types. **(A)** Higher expression of MAPK3 (*Mapk3)* in Endothelial cells **(B)** Higher expression of ERK2 (*Mapk1*) in Neutrophil cells. **(C)** Higher expression of MEK1 (*Map2k1*) in T cells **(D)** Higher expression of NF-κB (*Nfkb1*) in T cells **(E)** Higher expression of BAX (*Bax*) in Kupffer cells **(F)** Lower expression of BCL2 (*Bcl2*) in B cells.
