# Supplementary figures and images for: Bioinformatics analysis combined with experimental validation reveals the novel mechanisms of multi-targets of dapagliflozin attenuating diabetic liver injury
Source: Front Endocrinol (Lausanne). 2025 May 12;16:1519153. doi: 10.3389/fendo.2025.1519153 (PMC12117336; doi:10.3389/fendo.2025.1519153)

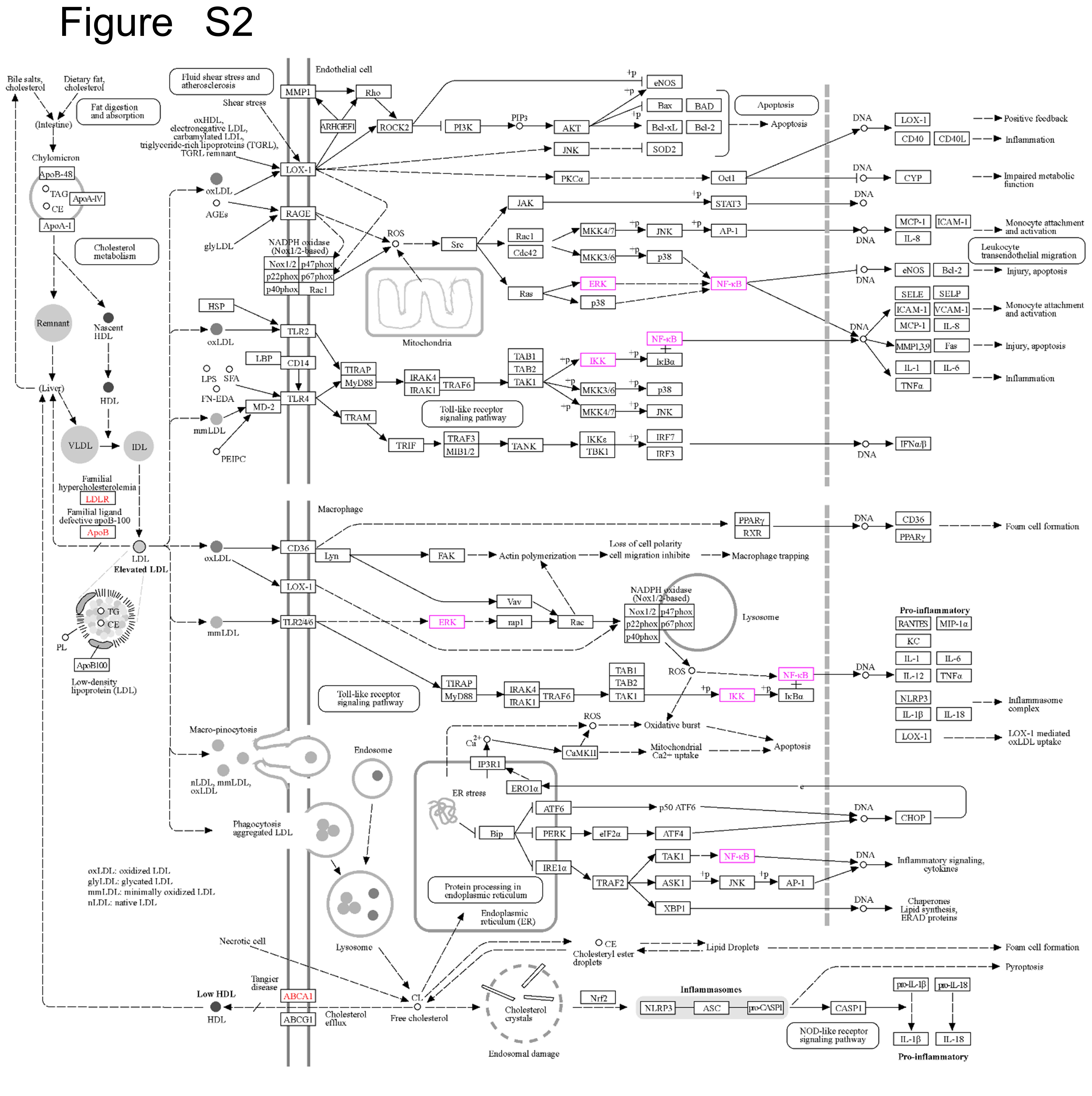

Supplement: Supplementary Figure 1 — (A) The volcano plot were display after the analysis of GSE2899, the blue and red dots represents the down-regulated and up-regulated genes, respectively. (B) The heatmap of top 10 down-regulated and up-regulated genes were conducted. (C) GS and MM analysis were constructed in MEbrown module. (D) The visualization of the eigengene network represents the relationship among the modules (MEbrown) and traits (diabetic liver injury). (E, F) The MCODE of overlapping genes of dapagliflozin and diabetic liver injury.The blue and red network represents the MCODE1 and MCODE2 network, respectively. The top 3 KEGG pathways of MCODE1 and MCODE2 were enriched in (F). [file SupplementaryFile1.zip › Supplementary Figures/Figure S2.TIF]
